# Degree of Landscape Fragmentation Influences Genetic Isolation among Populations of a Gliding Mammal

**DOI:** 10.1371/journal.pone.0026651

**Published:** 2011-10-28

**Authors:** Andrea C. Taylor, Faith M. Walker, Ross L. Goldingay, Tina Ball, Rodney van der Ree

**Affiliations:** 1 School of Biological Sciences, Australian Centre for Biodiversity, Monash University, Clayton, Australia; 2 School of Environmental Science and Management, Southern Cross University, Lismore, Australia; 3 Central Queensland University & Queensland Parks and Wildlife Service, Mackay, Australia; 4 Australian Research Centre for Urban Ecology, Royal Botanic Gardens Melbourne, c/- School of Botany, University of Melbourne, Parkville, Australia; Australian Wildlife Conservancy, Australia

## Abstract

Forests and woodlands are under continuing pressure from urban and agricultural development. Tree-dependent mammals that rarely venture to the ground are likely to be highly sensitive to forest fragmentation. The Australian squirrel glider (*Petaurus norfolcensis*) provides an excellent case study to examine genetic (functional) connectivity among populations. It has an extensive range that occurs in a wide band along the east coast. However, its forest and woodland habitat has become greatly reduced in area and is severely fragmented within the southern inland part of the species' range, where it is recognised as threatened. Within central and northern coastal regions, habitat is much more intact and we thus hypothesise that genetic connectivity will be greater in this region than in the south. To test this we employed microsatellite analysis in a molecular population biology approach. Most sampling locations in the highly modified south showed signatures of genetic isolation. In contrast, a high level of genetic connectivity was inferred among most sampled populations in the more intact habitat of the coastal region, with samples collected 1400 km apart having similar genetic cluster membership. Nonetheless, some coastal populations associated with urbanisation and agriculture are genetically isolated, suggesting the historic pattern observed in the south is emerging on the coast. Our study demonstrates that massive landscape changes following European settlement have had substantial impacts on levels of connectivity among squirrel glider populations, as predicted on the basis of the species' ecology. This suggests that landscape planning and management in the south should be focused on restoring habitat connectivity where feasible, while along the coast, existing habitat connectivity must be maintained and recent losses restored. Molecular population biology approaches provide a ready means for identifying fragmentation effects on a species at multiple scales. Such studies are required to examine the generality of our findings for other tree-dependent species.

## Introduction

Loss and fragmentation of habitat are recognised worldwide as substantial threats to biodiversity [1]–[3]. Habitat fragmentation can create small, isolated populations that are at increased risk of extinction through demographic and genetic stochasticity [4], [5]. Biodiversity management and planning will benefit from a thorough understanding of fragmentation impacts on a range of species [6]. However, the ways in which fragmentation of different origin and extent impact different species may not be readily predictable (see for example [7]–[9]). The relationship between landscape connectivity and functional connectivity for any given species will be determined by many interacting factors, including its feeding ecology, social organisation, predation risk, reproduction, vagility and corridor use [4], [10]–[12]. Therefore, to predict the impact of landscape fragmentation on functional connectivity would require data not only on each of these factors, but also on the expected net outcome of their interactions in a fragmented landscape [13]. For most species this level of knowledge has not yet been attained. Studies that test how well we can predict functional connectivity based on current knowledge of a species' ecology may therefore help improve and generalise predictive capacities.

Population genetic approaches provide a direct way of determining functional connectivity for a given species at the landscape scale. This is because the degree of genetic similarity among populations is determined largely by gene flow, which in turn reflects success in both dispersal among habitat remnants, and breeding [5]. The recent development of methods for using the information contained in a series of individual genotypes (rather than just gene pools and their allele frequencies) has revolutionised population genetics [14]–[16]. As well as providing higher resolution, these approaches mean that genetic affinities among individuals and groups of individuals can be examined at any spatial scale, without the need to pre-define population boundaries [17]. The same genotype data set can also be used to determine the extent to which small, isolated populations have lost genetic diversity, as well as providing a baseline for measuring the success of mitigation actions such as habitat restoration and corridor construction.

Among the most heavily human-impacted ecosystems worldwide are forests and woodlands, which are not only exploited for timber, but are cleared for agriculture because they typically occur on high fertility soils [6], [18]. Substantial losses of wooded habitat have occurred in Australia over its short history of European settlement; around half of the woodlands and forests have been modified or removed to make way for agricultural development, and only a small proportion of those remaining are protected [2], [19]. This has clear implications for the viability of the large number of forest and woodland dependent vertebrate species, particularly those requiring tree hollows (cavities) for shelter and breeding because these vital habitat resources are generally only present in century-old trees [20], [21].

Some hollow-using species with wide latitudinal ranges in eastern Australia are listed as threatened in only part of their range, promoting some indifference to a range-wide concern about their conservation. The squirrel glider (*Petaurus norfolcensis*) typifies this pattern, being listed as endangered in the south of its geographic range [22] but not-threatened in the north [23]. This pattern largely reflects differences in loss and fragmentation of habitat across a range that extends over 2700 km. Suitable habitat in the south, where squirrel gliders occur only inland of the Great Dividing Range, is now primarily restricted to a mosaic of fragmented and isolated patches of forest and woodland, many of which occur as linear strips along roadsides and as narrow riparian buffers. To what extent these linear remnants promote connectivity throughout the region is not known. Forest cover in this region has reduced by 65% since European settlement, and the habitat configuration present today has been established for over a century [24]. In the northern coastal parts of the range habitat fragmentation intensified in the last 20 years and has generally been less extensive than in the south [25], [26]. Nonetheless, the future of the squirrel glider in southeast Queensland is potentially not secure, because human population growth in the State as a whole, and in the southeast in particular, is outstripping that elsewhere in Australia [27], and suitable habitat for the squirrel glider is largely outside the reserve system [23], [25], [28].

The squirrel glider provides an excellent case study for understanding fragmentation effects over broad geographic scales. We predict that tree-dependent species such as the squirrel glider that rarely venture to the ground will be highly sensitive to forest fragmentation. Its mode of movement that involves gliding from tree to tree, means that gaps in tree cover greater than its gliding distance will limit its ability to move between habitat patches when foraging or dispersing, and if forced to move along the ground it may be exposed to high levels of predation [22], [29]–[32]. Here we analyse the landscape-scale distribution of nuclear (microsatellite) genotypic diversity in order to test the prediction that population genetic structure will be more pronounced in the south, where habitat clearing has occurred over a long period, than in coastal New South Wales and Queensland, where potential barriers to dispersal have become manifest only in recent decades. There is no geographic influence on squirrel glider ecology (see [33]–[35]) so we expect any differences between regions in genetic connectivity to be due to differences in habitat connectivity. We further predict that genetic diversity will be reduced in the south compared to the coastal region, as a consequence of isolation in small remnants. We discuss how the results increase our understanding of fragmentation impacts on the species and its viability, in the face of ongoing habitat alterations, and suggest how this may inform habitat restoration and planning that will benefit the species.

## Results

**Figure 1:**
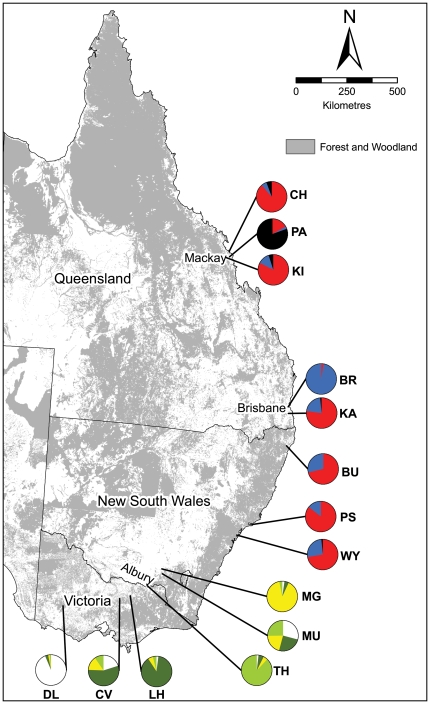
Location and STRUCTURE cluster membership (pie charts) of squirrel glider sampling sites in eastern Australia. Forest and woodland cover (National Vegetation Information System; Australian Government Department of the Environment and Water Resources, sourced 2005) is also shown. Site names are abbreviated as per Table 1.

Samples from 14 locations in a range of landscape contexts across the southern inland and coastal regions (Figure 1, Table 1) were genotyped at five microsatellite loci known to be highly variable in the species and accordingly displayed a large amount of allelic variation. The total number of alleles per locus across all populations was: 13 for Pn49, 24 for Pn16, 42 for each of Pet6 and Pet9 and 79 for Pet1. Genetic diversity parameters for each locus in each STRUCTURE-defined population (see below) are given in Table S1.

**Table 1:** Genetic diversity and differentiation parameters for thirteen squirrel glider populations defined by STRUCTURE groupings and sampling location (see text).

Population	Year of sampling	Abbrev	N	Hs	AR	Avge *F*_ST_ (within region)	Habitat and landscape context
SOUTHERN REGION							
Deep Lead Nature Conservation Reserve*	2004	DL	34	0.77	5.91	0.096	FB(1800)/A
central Victoria	2004	CV	15	0.85	8.19	0.075	L/A
Lurg Hills*	2004	LH	18	0.82	6.92	0.096	L/A
Thurgoona*	2003	TH	27	0.78	6.67	0.106	P
Murraguldrie State Forest	2001–2003	MU	24	0.86	8.02	0.070	FB(4500)/A
Mates Gully Travelling Stock Route*	2001–2003	MG	30	0.88	8.58	0.094	L/A
COASTAL REGION							
NSW central coast	2003	WY/PS	14	0.90	9.59	0.056	R
Bungawalbin Nature Reserve	2001	BU	6	0.90	7.60	0.058	FB(>5000)/R
Karawatha	2006–2008	KA	32	0.92	11.37	0.046	FB(750)/P
Bracken Ridge*	2003	BR	15	0.85	7.22	0.077	FB(140)/S
Kinchant Dam	2003–2005	KI	13	0.91	9.90	0.051	FB(480)/R
Cape Hillsborough	2003–2005	CH	10	0.91	9.39	0.069	R
Padaminka Nature Refuge*	2003–2005	PA	22	0.82	7.66	0.112	FB(64)/A

*Populations identified genetically as ‘isolates’. N = sample size, Hs = gene diversity and AR = allelic richness. For landscape context: FB = forest block (size in Ha); L = linear habitat along roadsides and rivers, with occasional small remnants and varying degrees of connectivity; P = peri-urban; S = suburban; A = agricultural land-use; R = rural (mixed land-use without extensive areas of agricultural development).

### Structure analysis

#### Southern region

**Figure 2:**
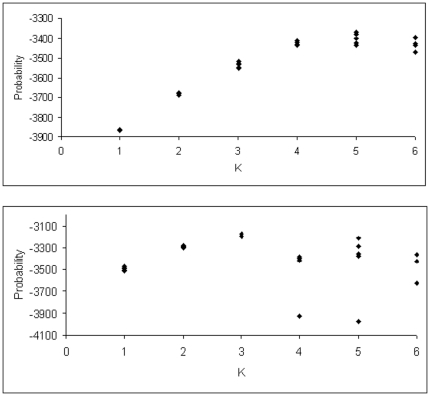
STRUCTURE likelihood values for different numbers of populations K. Five replicates are shown at each K in the southern (A) and coastal (B) squirrel glider samples.

STRUCTURE likelihood analysis suggested K = 4 as the most likely number of genetic clusters in the southern region samples (Figure 2a): at K = 5 no further geographic separation was introduced, and there was no clear or consistent increase in likelihood over that for K = 4. Four sampling sites had membership predominantly in a single cluster [Deep Lead in cluster 1 (Q = 0.94), Lurg Hills in cluster 2 (Q = 0.89), Thurgoona in cluster 3 (Q = 0.89) and Mate's Gully in cluster 4 (Q = 0.90)] suggesting isolation of those populations from others in the region (Figure 1). The other two sites had Q values more evenly distributed among clusters, suggesting they may be part of larger, more continuous populations in their region (Figure 1). The close proximity (∼25 Km) of Mate's Gully to Murraguldrie belies the low genetic connectivity between them, as to some extent does the proximity of Lurg Hills and the ‘central Victoria’ samples. The surrounding matrix in both these areas is largely cleared agricultural land with some roadside vegetation and scattered paddock trees.

#### Coastal region

The most likely number of clusters represented by the coastal region samples was 3; for K = 4 there was large variance in likelihood among replicate runs (Figure 2b) without the introduction of further geographically-based structure (e.g. no indication of separation of coastal NSW from Queensland sampling units; data not shown). The Padaminka sample had membership predominantly in cluster 1 (Q = 0.81), suggesting that this population, now surrounded by sugar cane plantations, currently experiences little or no immigration (Figure 1). Bracken Ridge (suburban Brisbane) had strong membership to cluster 2 (Q = 0.97; Figure 1). All other sampling sites, including the second Brisbane sampling site of Karawatha and pairs separated by as much as 1400 km straight-line distance, had Q values predominantly in cluster 3 (range 0.71–0.89; Figure 1). These results suggest the presence of broadscale connectivity over large distances in the coastal region, though this is now being impacted by habitat loss and fragmentation in some parts of the Brisbane and Mackay suburban and peri-urban areas. Because of their similar genetic cluster memberships (see Figure 1) and relatively close geographic proximity (around 70 km) we combined the Port Stephens and Wyong sample to increase the sample size for subsequent analyses (‘NSW central coast’ in Table 1).

### Pairwise relatedness within populations

**Figure 3:**
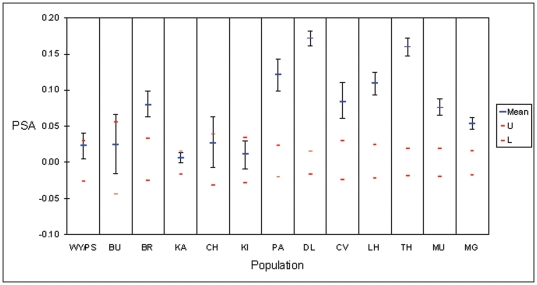
Mean proportion of shared alleles (PSA) as a measure of relatedness among individuals sampled from each population. A mean value that lies outside the 95% confidence interval bounded by the red lines indicates that relatedness for that population is elevated above that expected under the null hypothesis of no difference among populations.

All of the southern populations, along with Padaminka and Bracken Ridge from the coastal region, had significantly elevated mean relatedness (proportion of shared alleles, or PSA: Figure 3), suggesting that they received little input in the way of unrelated immigrants. This result is concordant with the identification based on STRUCTURE, of most of those populations (excluding Murraguldrie and central Victoria) as ‘isolates’, and suggests that discontinuous sampling is not driving that effect.

### Hardy-Weinberg and linkage disequilibria

Only two of the 65 locus/population tests revealed significant departure from Hardy-Weinberg equilibrium (both due to heterozygous deficits), after Bonferroni correction for multiple tests (Rice 1989): Pet9 in the Cape Hillsborough and Karawatha samples. This locus also showed heterozygous deficits in two other coastal populations based on pre-correction probability scores, suggesting the possible presence of null alleles at this locus in the coastal region. There was no indication of concerted linkage disequilibrium for any locus pair, and of 130 population/locus pair tests none were significant after correction for multiple tests.

### Genetic differentiation among STRUCTURE-defined populations

All pairwise *F*_ST_ values (Table 2) differed significantly from zero (P<0.03), indicating divergence in allele frequencies between all population pairs. However, non-urban populations in the coastal region (with the exception of the Padaminka isolate) were only minimally diverged from one another (*F*_ST_ values ranging from 0.021 to 0.057; Table 2), while non-urban southern populations had *F*_ST_ values ranging from 0.059–0.119 (Table 2). The populations at Bracken Ridge and Thurgoona (respectively suburban and peri-urban) had relatively high average *F*_ST_ values for their region, but the peri-urban site at Karawatha had the lowest average *F*_ST_ for the coastal region (Table 1). The populations identified as isolates had the highest average *F*_ST_ values in their region. This genetic divergence did not have a temporal component, as the isolates were sampled at similar times to other populations within their region (Table 1). Overall, southern populations were significantly more genetically divergent from one another than were coastal populations (Resampling Stats, P = 0.042). Euclidean distance and *F*_ST_ were not significantly correlated in either the coastal or southern regions (Mantel test, coast P = 0.36, south P = 0.37).

**Table 2:** Pairwise *F*_ST_ values (all significantly greater than zero; P<0.03) among STRUCTURE-defined squirrel glider populations (see Table 1).

	WY/PS	BU	BR	KA	CH	KI	PA	DL	CV	LH	TH	MU
BU	0.021											
BR	0.066	0.068										
KA	0.032	0.027	0.043									
CH	0.052	0.057	0.082	0.051								
KI	0.027	0.047	0.070	0.027	0.050							
PA	0.110	0.126	0.134	0.097	0.121	0.083						
DL	0.128	0.106	0.150	0.107	0.155	0.106	0.190					
CV	0.086	0.067	0.114	0.062	0.095	0.069	0.143	0.061				
LH	0.077	0.050	0.119	0.064	0.106	0.080	0.162	0.111	0.069			
TH	0.109	0.129	0.144	0.088	0.126	0.068	0.157	0.128	0.099	0.128		
MU	0.066	0.053	0.093	0.051	0.093	0.044	0.127	0.059	0.059	0.077	0.061	
MG	0.081	0.074	0.094	0.060	0.081	0.063	0.115	0.119	0.079	0.087	0.109	0.067

### Genetic diversity within populations

The lowest level of genetic diversity was seen in the western Victorian population of Deep Lead (Table 1). The Thurgoona, Lurg Hills, Padaminka and Bracken Ridge isolates also showed relatively low gene diversity (Hs) (Table 1). Overall, genetic diversity was higher in the coastal than southern populations, significantly so for allelic richness (P = 0.029) but not gene diversity (P = 0.070). The group of populations with high connectivity as defined by having Q values relatively evenly spread across multiple clusters (NSW central coast, Bungawalbin, Karawatha, Cape Hillsborough, Kinchant Dam, central Victoria and Murraguldrie) had significantly higher allelic richness (P = 0.006) and gene diversity (P = 0.025) than did the isolates (Bracken Ridge, Padaminka, Deep Lead, Lurg Hills, Thurgoona and Mate's Gully).

## Discussion

Knowledge of functional (genetic) connectivity among populations is essential for a complete understanding of the threats posed to biodiversity by human activities. This is particularly the case for species with broad geographic ranges because threats may vary geographically or may be obscured by geographic barriers or historical events (e.g. [36]–[40]). Our analyses have revealed significant structure among squirrel glider populations (regardless of geographic distance) and loss of genetic diversity in some populations. Four of the six sites sampled from the southern range, and two of the seven from the coastal range, each had membership predominantly in a single genetic cluster. By contrast one coastal cluster group was represented at sites dispersed over ∼1400 km in the central and northern part of the range. These findings are consistent with our prediction that this tree-dependent species will be sensitive to loss of tree cover and that populations centred on locations exposed to fragmentation for long periods will show more pronounced genetic sructuring. Conversely, populations located in regions with, or which until recently had, extensive tree cover, will exhibit gene flow over large spatial scales. Differences in genetic diversity and differentiation parameters between the two study regions may be a function of time as well as the spatial extent of habitat fragmentation. The value of our study is that it has examined genetic connectivity among populations of a species distributed over a distance of ∼2500 km. Few previous studies have attempted this across eastern and southern Australia (e.g. [41]). It highlights that fragmentation impacts are not consistent across a broad range but that severe impacts observed in one region may well be precursors to what can be expected elsewhere if appropriate remedial action is not taken.

### Patterns of genetic connectivity in the southern range

Squirrel glider populations in the inland woodlands of Victoria and southern NSW typically showed high levels of genetic differentiation, even over small distances (e.g. ∼25 km between Murraguldrie and Mate's Gully). This general pattern was indicated by the STRUCTURE analysis, and the fact that *F*_ST_ values were significantly higher overall among southern compared to coastal populations (with the exception of Padaminka). In addition, populations identified as ‘isolates’ by STRUCTURE showed significantly elevated levels of pairwise individual relatedness, consistent with them experiencing little or no immigration. The extensive clearing for grazing (more than a century earlier) of the species' preferred habitat in the south has meant that habitat is largely restricted to linear strips of remnant vegetation associated with roads, travelling stock reserves and waterways [32], [33]. There are no obvious broad-scale topographic or vegetation-type discontinuities in the landscape that would suggest populations might have been disjunct before habitat clearance. Furthermore, the fact that these microsatellite markers reveal population connectivity over large distances along the coast indicates that the strong nuclear genetic structure seen among most southern sampling sites is not a ubiquitous feature of contemporary squirrel glider populations but is likely a result of habitat clearing and fragmentation, which has resulted in population fragmentation. Low levels of population connectivity have also been observed in this region for another forest-dependent mammal, the yellow-footed antechinus (*Antechinus flavipes*) [42].

### Patterns of genetic connectivity in the coastal range

Two patterns were observed in the coastal range. The first was that samples from central NSW and central Queensland, separated by at least 1400 km, had similar genetic cluster membership, and the second, that two sample locations showed a high level of genetic isolation. The large extent of forest and woodland cover persisting in the coastal part of the species' range appears to have facilitated broad-scale connectivity, at least until recently: the extent to which the genetic signatures examined here will lag behind cessation of gene flow is not clear. The five locations with similar genetic cluster membership are embedded within, or closely connected to, broader areas of suitable habitat [26], [43]–[46]. For example, the broad area extending from the NSW-QLD border to north of Mackay retained approximately 50% forest and woodland cover in 1999 [26]. The two locations that showed genetic isolation (Padaminka near Mackay; Bracken Ridge in the city of Brisbane) have been isolated for at least 40 years. These sites presented evidence of genetic isolation as strong as those of remnants in Victoria, including elevated relatedness and reduced genetic diversity. By contrast, other sample locations around Mackay (Kinchant Dam; Cape Hillsborough) and Brisbane (Karawatha) are part of the broad-scale cluster group. Nonetheless, intensive agriculture and urbanisation have been shown to restrict movements of gliders in these areas in the last 20 years [30], [46] so we predict that this will be reflected in genetic measures of isolation in the future. Genetic isolation of koala (*Phascolarctos cinereus*) populations in urbanised areas of southeast Queensland has also recently been demonstrated [47], attesting to the significant impact that roads and housing can have on arboreal mammals.

### Loss of genetic diversity in isolated populations

We identified two populations in the coastal and four in the southern region showing signs of isolation, most likely as a consequence of urban and agricultural development. These six populations exhibit the loss of genetic diversity expected for small isolates, having significantly lower diversity than those populations that showed signs of connectivity with a broader population in their region. Loss of neutral genetic diversity as a result of habitat fragmentation was a consistent pattern observed in a meta-analysis performed by DiBattista (2008)[48].

The neutral genetic markers used in this study are unlikely to have a direct effect on fitness of individuals or populations. Whether genetic diversity at neutral markers reflects that in quantitative traits, and thus, their evolutionary potential, is unclear [49]–[51]. However, in relevant empirical studies of anthropogenically fragmented populations, fitness and/or quantitative trait variation were reduced along with neutral marker variation [52], [53]. It is therefore possible that the viability of the squirrel glider isolates identified in this study may be compromised by loss of genetic diversity, as well as demographic stochasticity. Comparison of fitness-related traits of animals within isolates and within continuous habitat should be investigated to determine whether loss of variation in neutral genetic markers is associated with reduced fitness.

### Implications for species viability, management and further research

We have identified two important characteristics of contemporary squirrel glider populations: i) genetic connectivity has persisted over large spatial scales where habitat is relatively continuous, and ii) fragmentation of habitat leads to genetic isolation. The latter was found for populations across the geographic range, suggesting that this reflects local impacts and is a general phenomenon. Our data are thus consistent with the suggestion that habitat fragmentation is associated with the regional decline of the species [54].

The vegetation-cover threshold at which squirrel glider population connectivity will be lost is not yet understood, but may become clear following fine-scale landscape genetics analyses that will be undertaken as our sample coverage improves (e.g. see [42], [55]). The results from such analyses will allow us to derive landscape design prescriptions that have the potential to improve habitat availability and connectedness for the squirrel glider. In addition, our baseline genetic variation and structure information will provide a powerful means of monitoring the success of mitigation programs [56] directed at the squirrel glider. These currently include the installation of rope canopy bridges across roads [57], [58] or tall wooden poles between habitat fragments [30], [59]. The scale at which these need to occur (in combination with revegetation programs) to reduce or prevent genetic isolation is not known, and should be complemented by genotypic approaches [56] to determine effective gene flow. Genetic analyses such as those described in this study need to be extended to other ground-avoiding arboreal mammals to determine whether this group of species is especially sensitive to genetic isolation.

## Materials and Methods

### Ethics statement

The samples were collected with institutional animal ethics approval, namely: Southern Cross University 02/2, 03/4, 03/10, 04/7 and 04/28; Central Queensland University 03/06–141; Deakin University (A05/96); and The University of Melbourne 02119, 05141, and 0810924.

### Study site details

Genetic samples from squirrel gliders were collected opportunistically as part of other studies on the species from 14 sites across eastern Australia, ranging from south-western Victoria, to around Mackay in central Queensland (see Figure 1). Gliders were sampled from a range of habitat (linear with or without small remnants; forest or woodland blocks) and landscape contexts (agricultural, suburban or peri-urban; multipurpose rural landscapes) scattered across a distance of approximately 2300 km (see Table 1 and Figure 1).

### Sample collection

Squirrel gliders were caught in traps placed on the trunks of trees (see [30], [33], [34], [45] for trapping and processing details) or removed from nest-boxes, between 2001 and 2008, though within any one region typically over a period of <3 years. Ear tissue samples (2 mm diameter) were taken from captured individuals and stored in 95%+ ethanol prior to DNA extraction. Because nest-box groups likely consist mainly of family members [60], we analysed only a single adult male and female from any given nest box to minimize the chance of sampling closely-related individuals, which would bias estimates of population allele frequencies and genotypic similarity. Sample sizes per location ranged from four to 34, and totalled 148 for the southern, and 111 for the coastal region (259 in total).

### Genotyping

Genomic DNA was extracted from ear tissue following Sunnucks & Hales [61]. Population samples were genotyped for five microsatellite loci: Pn16 and Pn49 (developed for squirrel gliders [62]) and Pet1, Pet6 and Pet9 (developed for the closely-related *P. breviceps* [63]) using conditions outlined in the relevant publication. Even in the small samples of squirrel gliders used in those studies, these markers had large numbers of alleles: 21, 15, 22, 25 and 25, respectively. Microsatellite alleles were detected by electrophoresis on 6% polyacrylamide sequencing gels and either autoradiography (Pn16, Pn49) or using a LI-COR Global IR2 two-dye DNA sequencer (model 4200; Petb1, Petb6 and Petb9).

### Genetic analyses

We employed the Bayesian clustering program STRUCTURE 2.1 [64] to identify genetic clusters within which genetic disequilibria are minimised, regardless of geographic location. We analysed the coastal and southern samples separately, since it has been shown that the two groups are experiencing ongoing phylogeographic separation, and accordingly split strongly into two separate STRUCTURE groups [65]. For each of the coastal and southern regions we performed five runs at each of K = 1 to 6 (Figure 2) in order to identify the most likely number of genetic clusters in the sample, using 100,000 burnins followed by 100,000 runs, using the correlated allele frequencies and admixture models. Clustering characteristics (specifically Q–the genetic cluster membership proportion of each sampling location) and geographic location were used in combination to identify groups of sampled individuals that could be considered ‘populations’ for the purpose of subsequent population genetic analyses.

To gain further insight into the degree of connectivity/isolation of a population, a measure of pairwise relatedness (proportion of shared alleles: PSA) among sampled individuals within that population was calculated using GENALEX. The probability that mean observed PSA for a population was greater than that expected under the null hypothesis of no difference among populations, was assessed by comparison with upper and lower 95% confidence intervals generated from 1000 random reshufflings of values among populations.

Deviations of genotype frequencies from those expected under Hardy-Weinberg equilibrium were tested for each locus in each population sample using the exact test as implemented in GENEPOP 3.2d [66]. We estimated linkage disequilibrium among all locus pairs in all populations, also using GENEPOP. We used FSTAT 2.9.3 [67] to calculate gene diversity (Hs) and allelic richness (AR). The latter is a measure of allelic diversity made using a rarefaction method that takes into account differences in sample sizes, in this case based on the smallest population sample size of six individuals [68]. The significance of differences in genetic diversity parameters among population groupings of interest was assessed with a permutation procedure, using FSTAT.

Pairwise *F*_ST_ values as a measure of genetic differentiation among populations were calculated, and their significance assessed by 1000 permutations, using FSTAT. These were used along with Euclidean distances among sampling sites to examine isolation-by-distance for the coastal and southern regions separately, via Mantel tests performed in GENEPOP using 100 permutations. The difference between the coastal and southern regions in pairwise intra-region *F*_ST_ values was assessed in a two-sided permutation test using 10000 resamplings in Resampling Stats 5.0.2, (http://www.resample.com [69]).

## Supporting Information

Table S1 **Genetic diversity parameters per locus and population for 13 STRUCTURE-defined squirrel glider populations (sample sizes in parentheses).**
(DOC)
