# Supplementary material for: Degree of Landscape Fragmentation Influences Genetic Isolation among Populations of a Gliding Mammal
Source: PLoS One. 2011 Oct 28;6(10):e26651. doi: 10.1371/journal.pone.0026651 (PMC3203874; doi:10.1371/journal.pone.0026651)
Supplement: Table S1 — Genetic diversity parameters per locus and population for 13 STRUCTURE-defined squirrel glider populations (sample sizes in parentheses). (DOC) [file pone.0026651.s001.doc]

# Table S1. Genetic diversity parameters per locus and population for 13 STRUCTURE-defined squirrel glider populations (sample sizes in parentheses).

|  | **Pn16** | **Pn49** | **Pet1** | **Pet6** | **Pet9** |
| --- | --- | --- | --- | --- | --- |
| **NSWcentcoast (14)** | | |  |  |  |
| **A** | 9 | 7 | 15 | 13 | 15 |
| **Ho** | 0.71 | 0.93 | 0.86 | 0.86 | 0.79 |
| **He** | 0.83 | 0.81 | 0.90 | 0.90 | 0.91 |
| **Bungawalbin (6)** | | |  |  |  |
| **A** | 7 | 5 | 8 | 9 | 9 |
| **Ho** | 0.67 | 1.00 | 0.83 | 1.00 | 0.67 |
| **He** | 0.85 | 0.75 | 0.78 | 0.86 | 0.88 |
| **Bracken Ridge (15)** | | |  |  |  |
| **A** | 6 | 6 | 11 | 9 | 10 |
| **Ho** | 0.93 | 0.87 | 0.87 | 0.93 | 0.87 |
| **He** | 0.80 | 0.77 | 0.86 | 0.85 | 0.85 |
| **Karawatha (32)** | | |  |  |  |
| **A** | 17 | 10 | 25 | 24 | 27 |
| **Ho** | 0.75 | 0.75 | 0.91 | 0.91 | 0.78 |
| **He** | 0.91 | 0.78 | 0.94 | 0.94 | 0.95 |
| **Hillsborough (10)** | | |  |  |  |
| **A** | 10 | 6 | 11 | 13 | 9 |
| **Ho** | 0.90 | 0.67 | 0.80 | 0.90 | 0.44 |
| **He** | 0.87 | 0.73 | 0.88 | 0.90 | 0.84 |
| **Kinchant (13)** | | |  |  |  |
| **A** | 11 | 7 | 17 | 14 | 10 |
| **Ho** | 0.77 | 0.58 | 0.92 | 1.00 | 0.58 |
| **He** | 0.88 | 0.84 | 0.92 | 0.90 | 0.81 |
| **Padaminka (22)** | | |  |  |  |
| **A** | 7 | 7 | 17 | 10 | 14 |
| **Ho** | 0.73 | 0.82 | 0.86 | 0.55 | 0.70 |
| **He** | 0.79 | 0.80 | 0.88 | 0.74 | 0.77 |
| **Deep Lead (34)** | | |  |  |  |
| **A** | 6 | 5 | 10 | 12 | 9 |
| **Ho** | 0.71 | 0.74 | 0.76 | 0.91 | 0.76 |
| **He** | 0.63 | 0.68 | 0.81 | 0.87 | 0.79 |
| **Central Victoria (15)** | |  |  |  |  |
| **A** | 6 | 6 | 15 | 14 | 12 |
| **Ho** | 0.60 | 0.73 | 0.93 | 0.73 | 0.87 |
| **He** | 0.74 | 0.76 | 0.90 | 0.80 | 0.88 |
| **Lurg Hills (18)** | |  |  |  |  |
| **A** | 7 | 6 | 11 | 13 | 9 |
| **Ho** | 0.83 | 0.72 | 0.89 | 0.94 | 0.72 |
| **He** | 0.82 | 0.78 | 0.83 | 0.84 | 0.73 |
| **Thurgoona (27)** | | |  |  |  |
| **A** | 5 | 5 | 18 | 13 | 7 |
| **Ho** | 0.67 | 0.81 | 0.93 | 0.78 | 0.59 |
| **He** | 0.73 | 0.71 | 0.90 | 0.84 | 0.63 |
| **Murraguldrie SF (24)** | | |  |  |  |
| **A** | 11 | 5 | 16 | 15 | 10 |
| **Ho** | 0.75 | 0.83 | 0.92 | 0.96 | 0.79 |
| **He** | 0.82 | 0.78 | 0.90 | 0.91 | 0.79 |
| **Mates Gully (30)** | |  |  |  |  |
| **A** | 10 | 9 | 20 | 13 | 13 |
| **Ho** | 0.83 | 0.87 | 0.97 | 0.90 | 0.90 |
| **He** | 0.85 | 0.86 | 0.92 | 0.78 | 0.90 |
